# MetClo: methylase-assisted hierarchical DNA assembly using a single type IIS restriction enzyme

**DOI:** 10.1093/nar/gky596

**Published:** 2018-07-09

**Authors:** Da Lin, Christopher A O’Callaghan

**Affiliations:** Wellcome Trust Centre for Human Genetics, Nuffield Department of Medicine, University of Oxford, Roosevelt Drive, Oxford OX3 7BN, UK

## Abstract

Efficient DNA assembly is of great value in biological research and biotechnology. Type IIS restriction enzyme-based assembly systems allow assembly of multiple DNA fragments in a one-pot reaction. However, large DNA fragments can only be assembled by alternating use of two or more type IIS restriction enzymes in a multi-step approach. Here, we present MetClo, a DNA assembly method that uses only a single type IIS restriction enzyme for hierarchical DNA assembly. The method is based on *in vivo* methylation-mediated on/off switching of type IIS restriction enzyme recognition sites that overlap with site-specific methylase recognition sequences. We have developed practical MetClo systems for the type IIS enzymes BsaI, BpiI and LguI, and demonstrated hierarchical assembly of large DNA fragments up to 218 kb. The MetClo approach substantially reduces the need to remove internal restriction sites from components to be assembled. The use of a single type IIS enzyme throughout the different stages of DNA assembly allows novel and powerful design schemes for rapid large-scale hierarchical DNA assembly. The BsaI-based MetClo system is backward-compatible with component libraries of most of the existing type IIS restriction enzyme-based assembly systems, and has potential to become a standard for modular DNA assembly.

## INTRODUCTION

Advances in biology and biotechnology have led to development of a variety of methods for assembly of large DNA constructs (1,2). Existing methods can be largely divided into two groups. Firstly, homology-based methods, which use long sections of overlapping sequence between fragments to specify the order of assembly, such as Gibson assembly and yeast- or *Bacillus subtilis*-based *in vivo* homologous recombination approaches (3–5). Secondly, restriction enzyme- or recombinase-based methods, which use defined enzyme-specific sequences to specify the order of assembly, such as Biobrick and Golden gate assembly (6–8). There are significant disadvantages to these approaches. The homology-based methods rely on long overlapping stretches of sequence between DNA fragments, which results in long sections of scar sequence being left between the fragments for a modular DNA assembly system. The enzyme-based methods generally leave shorter scar sequences defined by the enzyme recognition sequence (recombinase or Biobrick methods) or the adhesive end generated by the enzyme (type IIS restriction enzyme-based methods), but the DNA fragment to be assembled needs to be free of the recognition sequence of the enzymes used. An ongoing challenge is to achieve a balance between reducing sequence design constraints, such as the need to avoid forbidden enzyme recognition sequences in the DNA to be assembled, and reducing the unwanted scar sequence during a modular assembly process. Improving the flexibility of the assembly process to allow reusability of component parts is also a key objective.

Among the existing assembly methods, the type IIS restriction enzyme-based DNA assembly approach, such as Golden Gate assembly, is attractive for modular DNA assembly because circular double stranded DNA can be used as an input, it is a simple one-pot reaction, and it leaves a relatively small 3–4 bp scar between assembled fragments (8). The Golden Gate assembly method relies on the ability of type IIS restriction enzymes to cut DNA at a position outside their recognition sequence. DNA fragments to be assembled can be provided in plasmids and flanked by type IIS restriction sites facing the fragments, so that once released from the plasmid, the fragments no longer contain the restriction site. This enables one-pot assembly of DNA from plasmids using a mixture of a DNA ligase and a type IIS restriction enzyme. The order of assembly is determined by the user-defined sequences of the adhesive ends generated by the chosen type IIS restriction enzyme, and the minimal scar size between fragments depends on the length of the adhesive end.

MoClo-based systems were developed from the Golden Gate assembly system to provide hierarchical multi-stage assembly of DNA (9,10). With the MoClo approach, the assembly vector contains a second pair of type IIS restriction sites for an enzyme that is different from the enzyme used for assembly. This allows the second enzyme to be used to release the correctly assembled DNA, which can in turn be used as an insert in the next stage of the assembly. Alternating sequential use of two different type IIS restriction enzymes and antibiotic selection markers enables hierarchical assembly of large DNA fragments.

A major drawback of the type IIS enzyme-based DNA assembly method is the necessity to remove internal type IIS restriction sites from within the DNA fragments to be assembled. A minimum of two enzymes are required for basic hierarchical assembly, and three enzymes are required for other schemes such as multi-step linear addition of DNA parts (9).

Here we report the design of a fast and powerful hierarchical assembly method named MetClo which uses only one type IIS restriction enzyme in the assembly process. We built proof-of-principle MetClo systems for three commercially available type IIS enzymes BsaI, BpiI and LguI, and assembled multiple fragments in each system. We demonstrated the utility of the MetClo approach for rapid hierarchical assembly of large DNA fragments using the BsaI-based MetClo system to assemble a 218 kb DNA fragment from 28 × 7.7 kb fragments in two stages. In addition, we constructed a set of standard BsaI-based MetClo vectors for flexible DNA assembly that is backward compatible with existing part libraries.

## MATERIALS AND METHODS

### Plasmid construction

Plasmids were constructed by standard restriction enzyme/ligation-based cloning techniques. DNA fragments for cloning were generated by gene synthesis (Integrated DNA Technologies or Invitrogen), or by PCR with Q5 polymerase (NEB). For proof-of-principle modular assembly of ∼1 kb DNA from 4 fragments, the acceptor vector backbone is based on the ampicillin-resistant MoClo vector pICH47732 (9), and the donor plasmid backbone is based on a kanamycin-resistant vector built by replacing the ampicillin-resistant gene in pICH47732 with a kanamycin-resistant gene. For hierarchical assembly of a 218 kb DNA from 28 fragments, the acceptor vectors are based on gentamicin or kanamycin-resistant low copy BAC vector backbones with an F replication origin, and the donor plasmids are based on kanamycin-resistant low copy vector backbones with a p15a or F replication origin. For MoClo assembly of four different 54 kb fragments, the acceptor vectors are based on gentamicin-resistant low copy BAC vector backbone with an F replication origin. Plasmids for methylase overexpression were built on a kanamycin-resistant low copy BAC vector backbone. The methylase gene sequences were codon optimized for expression in *E. coli* based on the protein sequences from the REBASE (11): M.Osp807II (GenPept ID WP_083443746.1), M.Rsp7740I (AJW38159.1), M2.NmeMC58II (AAF41140.1), M.SacI (AAC97118.1) and M.XmnI (AAC44403.1). The methylase genes were under the control of the J23100 synthetic promoter (iGEM BBa_J23100), and flanked by a zeocin selection cassette and sequences homologous to the *E. coli* arsB locus to facilitate stable integration into the *E. coli* genome by recombineering. Template plasmids for methylase assays are based on ampicillin-resistant pICH47732. Plasmids in the standard MetClo vector set for BsaI-based modular assembly were built on ampicillin, kanamycin or chloramphenicol-resistant low copy vector backbones with a p15a or F replication origin. Plasmids constructed are listed in Supplementary Table S1, and plasmid sequences in Supplementary Data in genbank format.

### Generation of methylase expressing strains

*E. coli* strains with stable expression of specific methylases were generated by lambda-red recombineering. Briefly, DNA fragments containing the methylase transcription unit, zeocin selection marker and flanking sequences homologous to the arsB locus were generated by PCR using the corresponding methylase-expressing BAC plasmid as template. The fragments were used for recombineering in DH10B cells followed by zeocin selection to insert the methylase expression cassette into the arsB locus. Clones with correct chromosomal insertions were verified by PCR and sequencing. The methylase expressing strains can be stably maintained without zeocin selection. Linear construct sequences for recombineering (MOsp807II-zeo-arsb, M2NmeMC58II-zeo-arsb and MXmnI-zeo-arsb) are listed as supplementary data.

### Modular assembly

For proof of principle modular assembly of ∼1 kb DNA from 4 fragments by MetClo, assembly reactions were set up using 60 fmol of each donor plasmid prepared from normal DH10B cells, 60 fmol of assembly vector prepared from DH10B strains stably expressing compatible methylase, 1,000 U T4 DNA ligase (NEB), and 5 U BsaI (NEB) or BpiI (Thermo Fisher Scientific) or 2.5 U LguI (Thermo Fisher Scientific) in 20 μl 1× T4 ligase buffer (NEB). The reaction condition was: 37°C 15 min, followed by 45 cycles of 37°C 2 min plus 16°C 5 min, then 37°C 20 min, and 80°C 5 min. Assembly reactions were transformed into normal DH10B chemical competent cells, and plated on LB plates with AIX selection (ampicillin 100 μg/ml, IPTG 100 μM, X-gal 50 μg/ml) at 37°C overnight. White colonies were expanded and screened by restriction digestion using the corresponding restriction enzyme and by DNA sequencing.

For hierarchical assembly of a 218 kb DNA from 28 fragments by MetClo, the assembly was carried out in two stages. In stage one, the fragments were assembled, seven per group, using 15 fmol of each donor plasmid prepared from DH10B cells, 15 fmol assembly vector prepared from DH10B-M.Osp807II cells, 1000 U T4 ligase (NEB), and 5 U BsaI (NEB) in 20 μl 1× T4 ligase buffer (NEB). The reaction condition was the same as described above. The assembled samples were then drop-dialyzed against 50 ml dH_2_O using 0.05 μm mixed cellulose esters membrane discs (Millipore) at room temperature for 1 h. 5 μl of the dialyzed sample was transformed into 25 μl NEB 10-beta electrocompetent cells (NEB) by electroporation at 0.9 kV 100 Ω 25 μF using a Gene Pulser and 1 mm electroporation cuvettes (Bio-Rad). Following electroporation, 1 ml LB medium was added to the cells and incubated at 37°C 220 rpm for 1 h. Cells were plated on LB plates with GIX selection (gentamicin 2.5 μg/ml, IPTG 100 μM, X-gal 50 μg/ml) at 37°C overnight. Four white colonies were expanded and screened by restriction digestion using XhoI (NEB).

In stage two, the 218 kb DNA was assembled using protocols similar to stage one with the following modifications. 30 fmol of each donor plasmid and 15 fmol assembly vector were used in the assembly reaction. Following modular assembly and before the drop dialysis step, 10 U BsaI was added to the assembly reaction for incubation at 37°C for 45 min followed by heat inactivation at 80°C for 5 min. Transformed cells were plated on LB plates with KIX selection (kanamycin 30 μg/ml, IPTG 100 μM, X-gal 50 μg/ml). A total of 27 white colonies from two transformations were screened by PCR using primers detecting the junction between the second and the third 54 kb fragments (TACCCACGTGATTCACGCTG and ATCCTACAGACTCGCTGTGG). A subset of PCR-positive clones were screened by restriction digest using XhoI with standard agarose gel electrophoresis, and BsaI with pulsed-field electrophoresis using the CHEF II system (Bio-Rad).

The same set of 28 insert plasmids from the stage one MetClo assembly were assembled in sets of seven per group using MoClo vectors to compare the performance of MetClo with MoClo. The MoClo assembly was undertaken using a protocol analogous to the MetClo protocol described above, but with 15 fmol MoClo assembly vector prepared from DH10B cells in the one-pot assembly reaction. Six white colonies from each assembly were expanded and screened by restriction digest using XhoI.

## RESULT

### Principle of MetClo

When a type IIS restriction enzyme recognition site partially overlaps the recognition site of a specific methylase, then the methylase may methylate bases within the type IIS restriction site, and this methylation may block the restriction enzyme activity at this site and so switch the site off. We term the methylase that can switch off a type IIS restriction site that partially overlaps its own recognition site as a ‘switch methylase’ for the given restriction enzyme. The overlapping site we term a ‘methylation-switchable’ restriction site, and the whole process ‘methylation-switching’.

**Figure 1.**
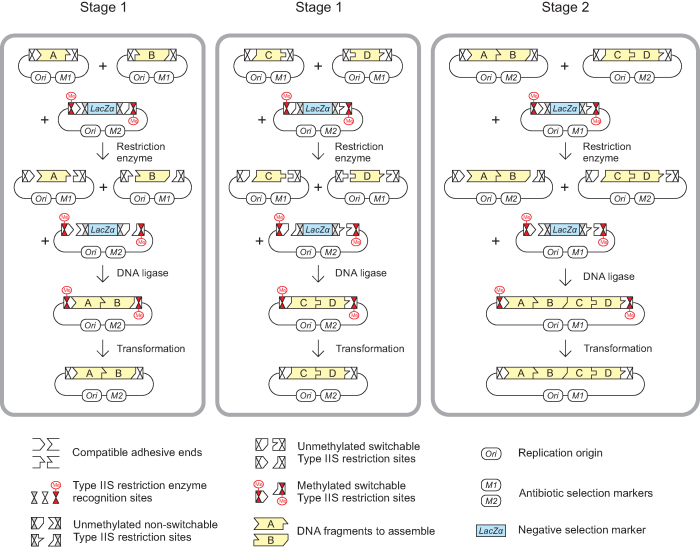
DNA assembly using MetClo. The figure illustrates the assembly of four DNA fragments (Fragments A, B, C and D, shown in yellow) into a single DNA fragment (Fragment ABCD) through two stages of MetClo using a single type IIS restriction enzyme. Stage 1 and Stage 2 are effectively the same process with progressively larger inserts. In Stage 1, the insert donor plasmids contain insert fragments (Fragments A, B, C or D) flanked by methylation-switchable type IIS restriction sites that generate compatible adhesive ends. Preparation of insert plasmids in normal *E. coli* strains, which lack the appropriate switch methylase, results in insert fragments that can be cut out by the type IIS restriction enzyme. The assembly vector contains the negative selection marker LacZα flanked by a purposely designed head-to-head configuration of type IIS restriction sites. The inner pair of restriction sites are designed to be non-switchable, but the outer pair are methylation-switchable, because the restriction enzyme recognition site overlaps with a recognition site for the switch methylase. Preparation of the assembly vector in *E. coli* strains expressing the switch methylase leads to selective methylation of the outer methylation-switchable restriction sites, and subsequent digestion with the type IIS restriction enzyme generates a vector backbone that retains the outer pair of methylated type IIS restriction sites. Ligation of the insert fragments into the vector backbone generates the correctly assembled plasmid, which is not cut by the type IIS restriction enzyme because these sites are still methylated. All of the other unwanted fragments including the vector backbone of the insert plasmid and the LacZα fragment from the assembly vector contain unmethylated type IIS restriction sites, which are cut by the restriction enzyme. This design allows a one-pot assembly reaction that combines the restriction and ligation step because the reaction favors generation of the correctly assembled plasmid. Transformation of the assembly reaction into a normal *E. coli* strain, which lacks the appropriate switch methylase, effectively results in demethylation of the methylated type IIS restriction sites flanking the assembled fragment in the assembled plasmid. In Stage 2, the assembled inserts (Fragments AB and CD) can then be used for a subsequent round of MetClo assembly into a larger fragment (Fragment ABCD).

MetClo uses methylation-switching to deliver a type IIS restriction enzyme-based one-pot DNA assembly system which allows the use of a single type IIS restriction enzyme throughout a hierarchical assembly process (Figure 1). Within the assembly vector there are a total of four type IIS restriction sites for the restriction enzyme used for assembly, with two sites arranged head-to-head on each flank of the negative selection marker. Of these four sites, the outer pair are methylation-switchable and the inner pair are non-switchable. Methylation of the assembly vector plasmid using the switch methylase selectively blocks the outer pair of methylation-switchable type IIS restriction sites, but leaves the inner pair unmethylated and so free to release the negative selection marker when cut by the type IIS enzyme. The one-pot assembly process favors replacing the negative selection marker with the correctly assembled insert fragments to generate a plasmid in which the assembled insert fragment is flanked by the outer switchable methylated type IIS restriction sites, because this plasmid is the only circular product that is not cut by the type IIS restriction enzyme. Transformation of the assembled plasmid into a standard *E. coli* strain that lacks the switch methylase results in plasmid that is not methylated at the insert-flanking methylation-switchable type IIS restriction sites. As a result, the assembled fragments can be released using the same type IIS restriction enzyme for the next stage of hierarchical DNA assembly (Figure 1).

### Identification of suitable methylase for methylation-switching

The key to the development of a practical MetClo system is identification of a site-specific methylase suitable for methylation-switching of the restriction site for the given type IIS restriction enzyme. This switch methylase must fulfill three essential criteria. Firstly, methylation of the overlapping methylation/restriction site must block the action of the type IIS restriction enzyme. Methylation could be achieved *in vitro* using purified methylase and vector plasmid, or by propagating the vector plasmid in a bacterial strain that expresses the methylase *in vivo*. Secondly, the methylase recognition sequence must not be identical to or entirely enclosed by the type IIS restriction site. This requirement arises because there are two sets of type IIS restriction sites for the same enzyme in the assembly vector: methylation-switchable and non-switchable (Figure 1). If the methylase recognition sequence is identical to, or enclosed by the type IIS restriction site, all the sites in the assembly vector for this restriction enzyme will be blocked by methylation; the resulting methylated assembly vector could not be cut by the restriction enzyme at all and so could not be used for DNA assembly. Thirdly, additional bases which specify the site of action of the methylase should not overlap with the adhesive end sequence generated by the type IIS restriction enzyme. This ensures maximum flexibility in the design of adapter sequences for DNA assembly.

We undertook a systematic search of REBASE for methylases with predicted ability to block restriction sites for the type IIS restriction enzymes BsaI, BpiI and LguI (11), which are common type IIS restriction enzymes used in hierarchical type IIS-based assembly systems (Table 1) (9,10,12–18). Desirable criteria were added to the essential criteria listed above to identify suitable candidates for experimental testing. To simplify vector preparation, we favored methylases that are active at the growth temperature of *E. coli* to allow *in vivo* methylation in strains constitutively expressing the methylase. We also preferred methylases that recognize longer sequences (≥5 bp), to reduce the number of bases within the bacterial host genome or assembly vector that would be collaterally methylated. This reduces the chance of an adverse effect on host gene function from *in vivo* methylation at other sites and could increase the efficiency of methylation by lowering the number of sites competing for the enzyme. In addition, we favored single subunit methylases that lack restriction activity for simplicity of transgene expression, and to avoid potential problems of restriction of plasmid DNA during vector transformation. Lastly, to increase the chance of methylation blocking the overlapping methylation/restriction sites, we preferred methylases that modify bases close to the middle of the restriction site.

**Table 1.** Type IIS restriction enzyme-based hierarchical assembly systems

Assembly system	Enzyme	Forbidden site frequency	Mode of assembly
MoClo (9,12)	BsaI, BpiI	1 in 1 kb	Multiplicative or iterative linear addition assembly^a^
GoldenBraid (10)	BsaI, BsmBI	1 in 1 kb	Multiplicative assembly
EcoFlex (13)	BsaI, BsmBI	1 in 1 kb	Multiplicative assembly
CIDAR (14)	BsaI, BpiI	1 in 1 kb	Multiplicative assembly
Yeast Toolkit (15)	BsaI, BsmBI	1 in 1 kb	Multiplicative assembly
TNT (16)	LguI, EarI	1 in 2 kb	Multiplicative assembly^b^
GreenGate (17)	BsaI	1 in 2 kb	Iterative linear addition assembly
PODAC (18)	BsaI	1 in 2 kb	Iterative linear addition assembly
MetClo-BsaI	BsaI	1 in 2 kb	Multiplicative or iterative linear addition assembly
MetClo-BpiI	BpiI	1 in 2 kb	Multiplicative or iterative linear addition assembly
MetClo-LguI	LguI	1 in 8 kb	Multiplicative or iterative linear addition assembly

^a^Three enzymes required for linear addition assembly.

^b^Maximum three parts per reaction with constraints in design of adaptor sequence.

**Figure 2.**
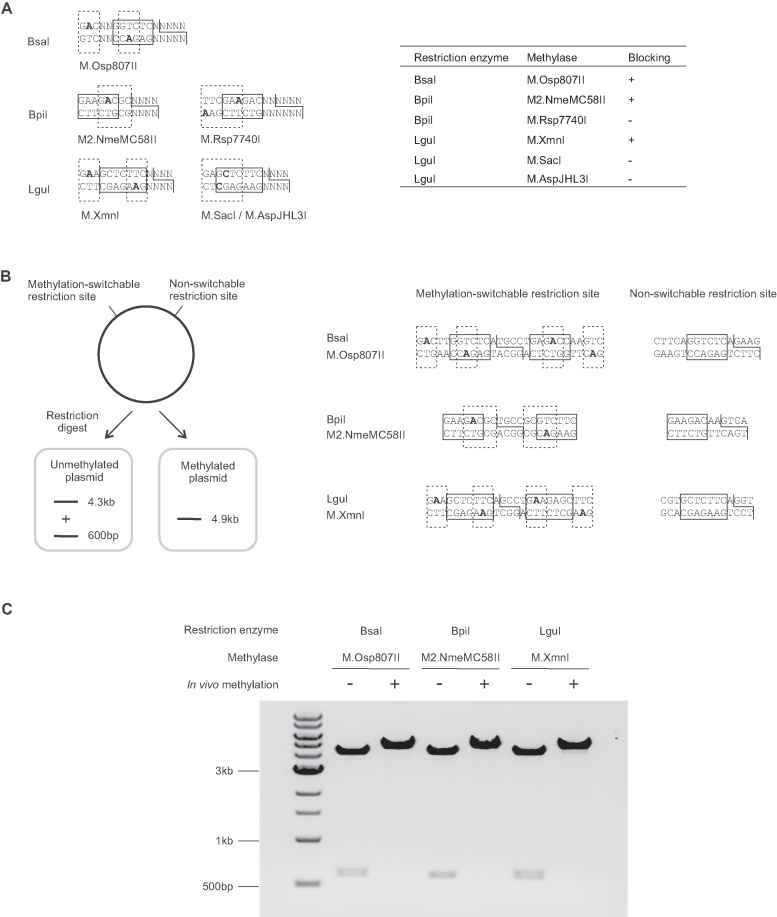
Identification of suitable methylases for methylation-switching. (**A**) Initial screening of functional methylases for selective blocking of overlapping methylation/restriction sites. The diagrams show the design of overlapping sites for screening of methylase activity. The table shows the screening result using methylases expressed *in vivo* from an F-ori based low copy number vector. Restriction enzyme recognition sites are boxed in solid lines. The adhesive ends generated by the restriction enzyme are shown by solid lines. Methylase recognition sites are boxed in dashed lines, and methylated bases are in bold font. All the listed methylases modify N6-adenine, except M.SacI and M.AspJHL3I, which modify C5-cytosine and N4-cytosine respectively. (**B**) Experimental designs to test blocking of methylation-switchable type IIS restriction enzyme sites by *in vivo* methylation. For each methylase/restriction enzyme combination tested, the test plasmid contains a head-to-head potentially methylation-switchable restriction site and a non-methylatable restriction site. Restriction digestion of test plasmid prepared from a normal *E. coli* strain would result in cutting at both sites and the release of a 600 bp fragment from the 4.3 kb vector backbone. Restriction digestion of test plasmid prepared from a strain expressing the switch methylase would result in a single 4.9 kb band, due to blocking of the methylation-switchable restriction sites by *in vivo* methylation. The restriction sites of the test plasmids for each restriction enzyme are shown, with the restriction site boxed in solid line, the methylase recognition site boxed in dashed line, and the methylated bases in bold. The head-to-head arrangement of overlapping methylation/restriction site allows the same assay to be used to detect any residual single strand nicking activity of the restriction enzyme towards the methylated restriction site. (**C**) Agarose gel electrophoresis analysis of the test plasmids for each methylation-switchable restriction site after preparation of the plasmids in a normal strain (–) or in a strain expressing the appropriate DNA methylase (+) and digested with the corresponding type IIS restriction enzymes. The combinations tested were BsaI with M.Osp807II methylase using test plasmid pMOP_BsaINC, BpiI with M2.NmeMC58II methylase using test plasmid pMOP_BpiINC, and LguI with M.XmnI methylase using test plasmid pMOP_LguINC. Test conditions were 60 fmol test plasmid digested using 5 U BsaI or BpiI, or 2.5 U LguI in 10 μl reactions at 37°C for 1 h. The results show that *in vivo* methylation by each of the methylases successfully blocked the restriction site for the corresponding type IIS restriction enzyme when the methylase recognition site overlapped the restriction enzyme site. The data shown represents results from three independent experiments.

Based on these criteria, we selected the following methylases for testing of methylation-switching activity: M.Osp807II for BsaI; M.Rsp7740I (19) and M2.NmeMC58II (20) for BpiI; M.SacI (21), M.AspJHL3I (22) and M.XmnI (23) for LguI (Figure 2A). We screened methylase activity by preparing ColE1-based high copy number template plasmids carrying overlapping methylation/restriction sites in DH10B cells expressing the corresponding methylase under the J23100 constitutive synthetic promoter from a low copy number BAC vector. This identified M.Osp807II (for BsaI), M2.NmeMC58II (for BpiI) and M.XmnI (for LguI) as suitable candidates for further analysis (Figure 2A). For each of these methylases, we integrated the methylase expression cassette with a zeocin selection marker into the arsB locus (24) using lambda-red recombineering to generate *E. coli* strains that constitutively overexpress the methylase from a chromosomal copy.

We tested the methylation-switching activity of these methylases *in vivo* using these strains. Test vectors were constructed that carry one restriction site that does not overlap with the methylase recognition sequence and so is non-switchable. In addition, these vectors include an element ∼600 bp away with two type IIS restriction sites in a head-to-head arrangement, both of which overlap with the corresponding methylation sequence and so may be methylation-switchable. The non-switchable site should be cut by the type IIS restriction enzyme regardless of methylase activity. The methylation-switchable sites should be blocked if the plasmid is generated in the strain expressing the switch methylase (Figure 2B). The head-to-head arrangement of these methylation-switchable sites also allows for assessment of any residual single-strand nicking activity by the type IIS restriction enzyme when the site is methylated; because these sites share a common cutting site, single-strand nicking activity at these sites will result in double-stranded cleavage similar to that seen with full restriction activity in the absence of any methylation. Restriction digest of test vectors prepared in normal strains or in strains expressing the switch methylase shows that *in vivo* expression of each of three switch methylases selectively blocks restriction digestion of the corresponding overlapping sites. This suggests that these methylases can be used for methylation-switching of sites for the corresponding type IIS restriction enzymes *in vivo* (Figure 2C).

### MetClo-based DNA assembly

**Figure 3.**
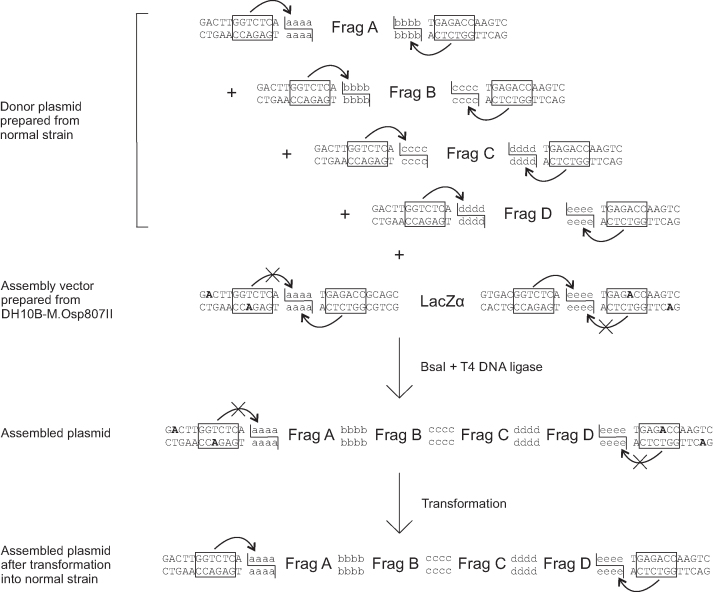
BsaI-M.Osp807II based MetClo system. The donor plasmids contain DNA fragments to be assembled (Fragments A–D) flanked by BsaI sites that generate compatible adhesive ends (schematically labelled aaaa-eeee). The BsaI sites overlap with the M.Osp807II methylase recognition sequence. Donor plasmids were prepared from a normal strain that does not express the M.Osp807II switch methylase. As a result, the BsaI sites are not methylated and so the insert DNA fragments can be released by BsaI digestion. The recipient assembly vector contains a LacZα selection marker flanked by head-to-head BsaI sites. The outer pair of BsaI sites closer to the vector backbone overlap with an M.Osp807II methylation sequence and so are methylation-switchable, whereas the inner pair of BsaI sites are not. Preparation of the assembly vector in the M.Osp807II switch methylase-expressing DH10B strain results in selective blocking of the outer pair of BsaI sites. The LacZα fragment can be released by BsaI through cutting at inner pair of BsaI sites. Following a one-pot reaction using BsaI and T4 DNA ligase, ligation among compatible adhesive ends results in ordered assembly of DNA fragments into the assembly vector backbone. The assembled fragment in the assembled plasmid is flanked by methylated BsaI sites, which are not cut by BsaI. Following transformation into a normal strain that does not express the M.Osp807II switch methylase, methylation of the flanking restriction sites is lost, and the assembled fragment can be released by BsaI for the next stage assembly.

We developed proof-of-principle one-pot MetClo DNA assembly systems for each of these three methylase/type IIS restriction enzyme combinations. As an example, Figure 3 illustrates the assembly of four DNA fragments by the BsaI-based MetClo system using *in vivo* methylation-switching by the M.Osp807II switch methylase. The assembly is designed so that the insert fragments to be assembled and the final assembled fragment are flanked by the same methylation-switchable BsaI sites. The insert plasmids are prepared in a standard *E. coli* strain that lacks the M.Osp807II switch methylase, so that the BsaI sites are not methylated and this allows BsaI to be used to cut the insert DNA out of the plasmid. The assembly vector is prepared in an *E. coli* strain that does express the M.Osp807II switch methylase, so that the switchable outer pair of BsaI sites are methylated and so cannot be cut by BsaI; they are switched ‘off’. Following one-pot assembly and transformation into a standard *E. coli* strain that does not express the switch methylase, the assembled plasmid is not methylated at the methylation-switchable BsaI sites that flank the assembled fragment, so this plasmid can then be cut by BsaI at these sites to release the assembled fragment for a further round of DNA assembly.

We tested the assembly of four fragments using the MetClo approach with each of the three enzymes BsaI, BpiI and LguI and the corresponding methylases (Table 2). The donor plasmids were kanamycin-resistant, and the assembly vectors were ampicillin-resistant. Assembly vectors were prepared in the corresponding switch methylase-expressing strains. Following selection, over 99% of the colonies were white (LacZ-negative). For each assembly, eight white colonies were analyzed by restriction digest and Sanger sequencing. These analyses established that 100% of the white clones were correct for each of the three MetClo systems (Table 2 and Supplementary Figure S1). This demonstrates that the MetClo approach can be used to assemble multiple DNA fragments using a single type IIS restriction enzyme in a one-pot reaction, resulting in a plasmid from which the assembled insert can be released using the same type IIS restriction enzyme.

**Table 2.** Proof-of-principle DNA assembly using BsaI, BpiI or Lgui-based MetClo systems

Plasmid	Vector	Inserts				Methylase	Enzyme	Size	Success rate
pMXP_test	pMXP_A4E	pMXK2_FragA	pMXK2_FragB	pMXK2_FragC	pMXK2_FragD	M.Osp807II	BsaI	908 bp	100% (8/8)
pMYP_test	pMYP_A5E	pMYK2_FragA	pMYK2_FragB	pMYK2_FragC	pMYK2_FragD	M2.NmeMC58II	BpiI	1152 bp	100% (8/8)
pMZP_test	pMZP_P6T	pMZK2_FragA	pMZK2_FragB	pMZK2_FragC	pMZK2_FragD	M.XmnI	LguI	1149 bp	100% (8/8)

To explore the utility of MetClo for hierarchical assembly of large DNA constructs, we assembled a 218 kb non-repetitive DNA fragment from 28 × 7.7 kb fragments using BsaI-based MetClo in two stages. In stage one, four sets of seven fragments were assembled into four intermediate fragments of ∼54 kb each using BsaI-based MetClo. In stage two, the 4 × 54 kb intermediate fragments were assembled into a single 218 kb fragment, again using BsaI-based MetClo. Following antibiotic selection, the percentage of clones that are LacZ-negative white clones was ∼5% for the stage one assembly, and ∼0.2% for the stage two assembly. The reduced percentage for stage two reflects the reduced transformation efficiency of the large assembled plasmid compared to that of the smaller uncut assembly vector. Of the white colonies screened, restriction digestion demonstrated a ∼50% success rate for stage one assembly, and ∼20% for stage two assembly (Table 3 and Supplementary Figure S2). To assess MetClo in comparison to standard MoClo, the same fragments were assembled using one-pot MoClo. The percentage of white clones for MoClo assembly was ∼5%, similar to MetClo. The success rate of MoClo assembly as assessed by restriction digestion of white clones was also similar to stage one assembly by MetClo (Supplementary Table S2). This demonstrates that the performance of MetClo is comparable to that of MoClo. These data confirm that the MetClo system can be used for hierarchical multi-step assembly of a large DNA construct using a single type IIS restriction enzyme.

**Table 3.** Hierarchical assembly of a 218 kb DNA fragment using a BsaI-based MetClo system

Plasmid	Vector	Inserts							Size	Success rate^a^
pMXBG_3A1	pMXBG_A7I	pMOLK_2A1	pMOLK_2B1	pMOLK_2C1	pMOLK_2D1	pMOLK_2E1	pMOLK_2F1	pMOLK_2G1	54 kb	75% (3/4)
pMXBG_3I1	pMXBG_I7G	pMOLK_2I1	pMOLK_2A2	pMOLK_2B2	pMOLK_2C2	pMOLK_2D2	pMOBK_2E2	pMOLK_2F2	55 kb	50% (2/4)
pMXBG_3G1	pMXBG_G7F	pMOLK_2G2	pMOLK_2I2	pMOLK_2A3	pMOLK_2B3	pMOLK_2C3	pMOBK_2D3	pMOLK_2E3	54 kb	50% (2/4)
pMXBG_3F1	pMXBG_F7E	pMOLK_2F3	pMOLK_2G3	pMOLK_2I3	pMOLK_2A4	pMOLK_2B4	pMOLK_2C4	pMOLK_2D4	54 kb	50% (2/4)
pMXBK_4A1	pMXBK_A7E	pMXBG_3A1	pMXBG_3I1	pMXBG_3G1	pMXBG_3F1				218 kb	20%^b^

^a^Calculated based on the white colonies selected following blue-white screening.

^b^14/27 verified by PCR screening, of which 3/8 verified by restriction digest.

### A standard MetClo modular assembly vector set

**Figure 4.**
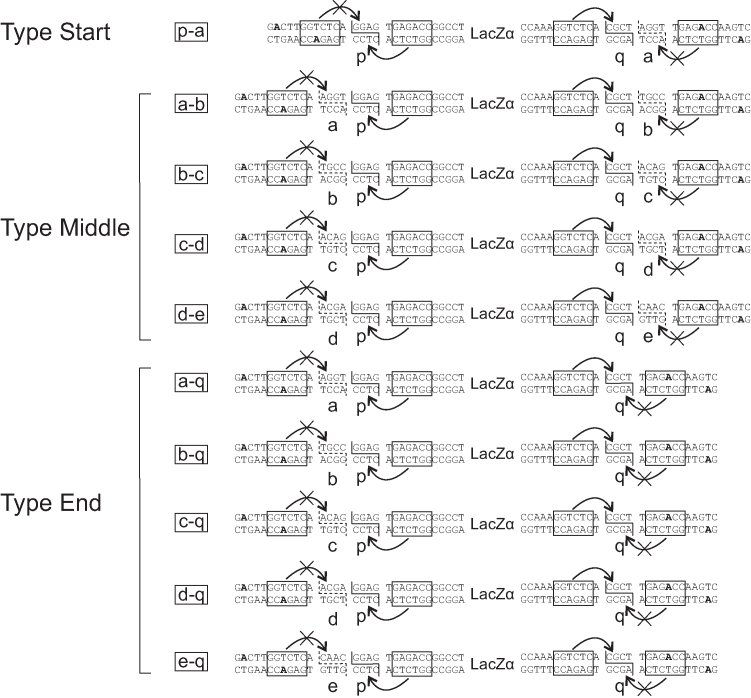
Design of a standard MetClo vector set. Three types of adaptor sequences were designed for the standard MetClo vector set. The vectors contain two head-to-head BsaI sites (boxed) flanking the negative selection marker LacZα. The pair of unmethylated BsaI sites closest to the negative selection marker can be used to release LacZα and generates adhesive ends ‘p’ and ‘q’. This allows any fragments that start with adhesive end ‘p’ and end with adhesive end ‘q’ to be cloned into any of the three types of adaptor sequences (types ‘Start’, ‘Middle’, ‘End’). The outer pair of BsaI sites closest to the vector backbone overlap with the M.Osp807II recognition sequence and are both methylated at the adenine bases highlighted in bold when the vector is prepared in M.Osp807II-expressing *E. coli* strain. A DNA fragment assembled into these vectors, following transformation into a normal *E. coli* strain which lacks this switch methylase activity, can be released from the assembled plasmid using BsaI, which can now recognize this unmethylated BsaI site. The released fragment will carry different adhesive ends depending on the type of the vector used. Assembly into a type ‘Start’ vector will generate a fragment flanked by adaptors ‘p-a’; assembly into a type ‘Middle’ vector will generate a fragment flanked by ‘a-b’, ‘b-c’, ‘c-d’ or ‘d-e’; assembly into a type ‘End’ vector will generate a fragment flanked by ‘a-q’, ‘b-q’, ‘c-q’, ‘d-q’ or ‘e-q’. The design of these LacZα selection cassettes flanked by unique adaptors are represented in the figure by the letter codes for the outside adaptor sequences.

**Figure 5.**
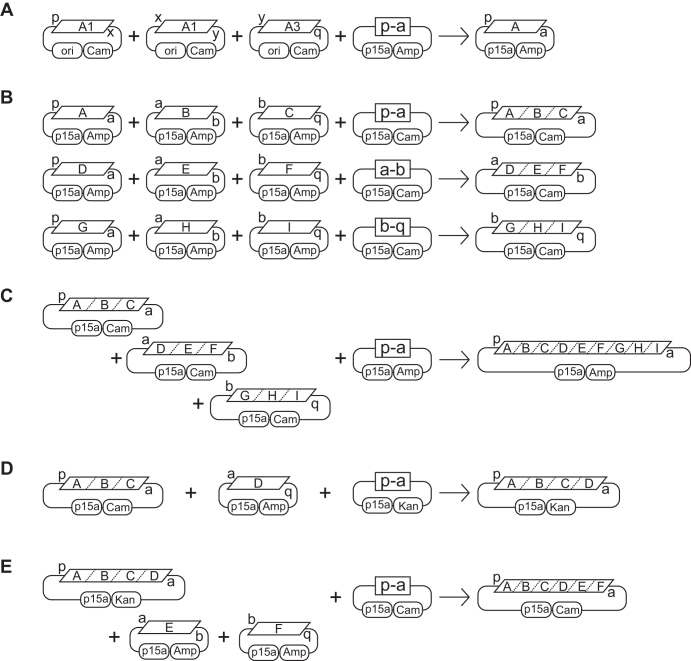
Schemes for DNA assembly using standard MetClo vector set. The MetClo vector set supports both multiplicative and linear additive topology for DNA assembly. (**A**) As an example, a hypothetical transcription unit ‘A’ can be assembled from a promoter fragment ‘A1’ that starts with adaptor sequence ‘p’ and ends with adaptor sequence ‘x’, a coding region fragment ‘A2’ that starts and ends with adaptor sequences ‘x’ and ‘y’, and a transcription terminator fragment ‘A3’ that starts and ends with adaptor sequences ‘y’ and ‘q’. The internal adaptor sequences ‘x’ and ‘y’ used to link the DNA parts in order at this stage are arbitrary adaptor sequences not specified by the MetClo vector set. Any compatible DNA parts can be assembled into the vector as long as the first part start with adaptor sequence ‘p’ and the last part end with adaptor sequence ‘q’. MetClo assembly of these parts into MetClo vector ‘p-a’ generates assembled transcription unit ‘A’ flanked by adaptor sequence ‘p’ and ‘a’. (**B**) With a multiplicative assembly scheme, assembled transcription units can be assembled three per group in different MetClo vectors into larger fragments that contain three transcription units ‘ABC’, ‘DEF’ or ‘GHI’. (**C**) These three fragments, ‘ABC’, ‘DEF’ and ‘GHI’, can then be assembled into a single fragment containing 9 transcription units. (**D**) Alternatively, additional transcription units can be added to an existing fragment containing multiple transcription units using a linear addition assembly scheme. Addition of transcription unit ‘D’ flanked by adaptors ‘a’ and ‘q’ to fragment ‘ABC’ flanked by adaptors ‘p’ and ‘a’ using assembly vector ‘p-a’ generates an assembled fragment ‘ABCD’ which is still flanked by adaptors ‘p’ and ‘a’. (**E**) Further addition of fragments ‘E’ and ‘F’ can be undertaken to generate a larger fragment ‘ABCDEF’.

Because MetClo uses a single type IIS restriction enzyme throughout DNA assembly, DNA parts with compatible adhesive ends from different stages of the assembly can be assembled with suitable assembly vectors. This provides great flexibility in assembly design. To exploit this feature, we designed and built a set of standardized MetClo assembly vectors for flexible modular DNA assembly (Figure 4 and Supplementary Figure S3). Each of these vectors can accept any DNA fragment which carries compatible adhesive ends, such that the first component fragment in the assembled sequence starts with adhesive end ‘p’, and the last component fragment in the assembled sequence ends with adhesive end ‘q’ (Figures 4 and 5A). The adhesive ends carried by the assembled DNA fragment depend on the choice of assembly vectors in the assembly process. Three types of assembly vectors were designed: Firstly, an assembly vector of type ‘Start’ generates assembled fragments that start with adhesive end ‘p’, and end with adhesive end ‘a’. Secondly, an assembly vector of type ‘Middle’ generates assembled fragments that start and end with adhesive ends ‘a-b’, ‘b-c’, ‘c-d’ or ‘d-e’. Thirdly, an assembly vector of type ‘End’ generates assembled fragments that start with adhesive end ‘a’, ‘b’, ‘c’, ‘d’ or ‘e’, and end with adhesive end ‘q’ (Figure 4). The adhesive ends designed for orderly multi-fragment DNA assembly were chosen based on a previous design to minimize cross-reactivity between non-compatible adhesive ends (25). We have designed the adhesive ends ‘p’ and ‘q’ such that the vector set is compatible with several existing part libraries for mammalian and plant synthetic biology (10,12,26), as well as the OpenPlant synthetic biology standard (27). The design of further adhesive ends would allow the assembly of more fragments in a single reaction if required.

DNA fragments assembled into type ‘Start’ assembly vectors can be used as the first fragment, fragments assembled into type ‘Middle’ as middle fragments, and fragments assembled into type ‘End’ as the last fragment for subsequent stages of DNA assembly (Figure 5A–E). Appropriate use of type ‘Middle’ and type ‘End’ vectors enables the assembly of between 2 and 6 fragments in each assembly reaction. Vector backbones with two different replication origins were designed to assemble both small and large fragments, and three different antibiotic selection markers were used for assembly of DNA fragments encoded in vectors that carry any two different selection markers (Supplementary Figure S3, Figure 5D and E). The resulting standard assembly vector set can be used to assemble an arbitrary number of standard DNA blocks (such as transcription units) in any order in the same orientation, as long as the standard blocks carry the same antibiotic selection marker and start and end with the same adhesive end ‘p’ and ‘q’. Up to 5 standard blocks can be added to any intermediate fragments that have been assembled in type ‘Start’ vectors. The vector set thus supports both multiplicative (Figure 5B and C) and linear additive (Figure 5D and E) assembly topologies.

## DISCUSSION

We have developed and tested a system for one-pot assembly of a DNA fragment from multiple donor plasmids using a single type IIS restriction enzyme. The assembled fragment can be released from the recipient plasmid by the same restriction enzyme, so allowing hierarchical assembly of large DNA constructs using this restriction enzyme again. This reuse of the same enzyme is possible because the system uses a site-specific switch methylase to regulate the activity of the restriction enzyme at a methylation-switchable restriction site. A strain expressing the switch methylase is used to prepare the assembly vector to provide *in vivo* methylation of methylation-switchable restriction sites within the vector. The one-pot assembly process is straightforward and preserves the simplicity of the existing type IIS restriction enzyme-based assembly systems, which do not use methylation-switching (Table 1). Standard unmodified *E. coli* cloning strains are used for transformation of the assembly reaction and preparation of donor plasmids containing the inserts to be assembled. The MetClo approach has a number of advantages over existing hierarchical DNA assembly systems.

Firstly, the need to remove internal restriction sites from component DNA fragments and plasmids is reduced by the use of a single enzyme. Compared with the standard MoClo system using two or more enzymes, the BsaI- and BpiI-based single enzyme MetClo systems each use one restriction enzyme that recognizes 6 bp and so reduces the frequency of forbidden restriction sites from 1 per 1 kb to 1 per 2 kb for random DNA with 50% GC content, and the LguI-based MetClo system uses a 7 bp cutter that reduces the frequency further to 1 per 8 kb (Table 1). The considerable improvement brought about by the LguI-based MetClo system potentially allows semi-synthetic construction of DNA component fragments by PCR with relatively minimal need for modification; this approach might be suitable for large-scale construction and characterization of libraries of native DNA elements such as promoters and enhancers. Further improvements to reduce the need for internal restriction site removal may depend on the discovery or development of new type IIS restriction enzyme with added specificity.

Secondly, MetClo increases the design flexibility of hierarchical assembly. The use of a single enzyme allows the assembly of DNA fragments that have been produced during different stages of a hierarchical DNA assembly. This enables the simple design of a vector set that is capable of both multiplicative and linear additive DNA assembly (Figure 5). The linear addition capability of the MetClo vector set is superior to existing systems such as MoClo. In MoClo, linear addition requires the use of three type IIS restriction enzymes and two different negative selection markers, and added DNA fragments form part of the vector backbone of the assembly vector for next stage assembly. This may pose problems for linear addition of DNA to existing large fragments in very low copy number vectors, because the low copy replication origin is necessary for maintenance of the large fragment in the vector backbone, but may also result in low and potentially inadequate expression of the selection marker carried by the assembly vector. With the MetClo vector set, however, the linear addition step is the same as multiplicative assembly in that a single negative selection marker is required and the marker always lies in a standardized vector backbone. Assembly vectors with a very low copy number vector backbone can be prepared as high copy number vectors using a high copy replication origin located close to the negative selection marker. These features therefore ensure robustness and simplicity for the negative selection step during linear additive assembly using the MetClo vector set. In addition, there are many possible permutations based on the MetClo system that could be added to expand the functionality of the standard vector set. This includes the option to assemble DNA fragments through an intermediate assembly stage, which is useful for subassembly of complex parts, such as multi-domain open reading frames, for added reusability of intermediate parts (Supplementary Figure S4). Another option is a standard method to incorporate methylation-switchable BsaI sites into existing vector backbones, which is useful for modular construction of common assembly vectors carrying functional elements within the vector backbones from existing functional modules (Supplementary Figure S5).

Thirdly, MetClo improves the exchangeability of component parts between different type IIS-restriction enzyme based part libraries. Two major barriers impede the exchange of DNA component parts between different libraries: the choice of different adaptor sequences for common classes of genetic elements, and the choice of different restriction enzymes for hierarchical DNA assembly. The former can be addressed by the use of common sequences or ‘syntax’ for equivalent classes of functional genetic elements as done in plant synthetic biology (27), but the latter results in significant sequence constraint to avoid unwanted cleavage at unintended restriction sites in component fragments. The independent development of component libraries and assembly standards has led to assembly systems that use different restriction enzymes (Table 1). It is difficult to develop common exchangeable DNA components that use different type IIS restriction enzymes, because this requires the removal of internal restriction sites for at least three enzymes, which together occur at a frequency of 1 in ∼700 bp. The MetClo system uses a single type IIS restriction enzyme and is compatible with any system that uses that restriction enzyme. In particular, the BsaI-based system is compatible with existing systems that use BsaI, which include most of the type IIS restriction enzyme-based assembly systems developed to date. The adaptor sequence design of the standard MetClo vector set we developed is compatible with existing part libraries for mammalian and plant synthetic biology (10,12,26), and BsaI-based part libraries for *E. coli* and yeast synthetic biology (13–15) can be assembled using the MetClo method by designing assembly vectors with suitable adaptor sequences. Therefore, the BsaI-based MetClo system has potential to be adopted as a standard for modular assembly that would be compatible with most existing component part libraries across different species.

There are existing DNA assembly systems that use DNA methylation to block internal type IIS restriction sites during the assembly process. The TNT assembly system uses two type IIS restriction enzymes, the 7 bp cutter LguI (GCTCTTCN∧NNN) and the 6 bp cutter EarI (CTCTTCN∧NNN) (16). Both enzymes generate 3bp sticky ends, and there is a requirement to remove the 6 bp sequence (CTCTTC) from DNA parts. The system uses the M.TaqI methylase to methylate an overlapping EarI recognition site within the assembly vector during assembly stages with EarI. However, because the M.TaqI recognition sequence (TCGA) that overlaps with the EarI restriction site intrudes into the adhesive end sequence (CTCTTCG∧ANN), the system placed substantial constraints on the adaptor sequences that can be used and so can only assemble three fragments at a time. Other examples include Greengate and PODAC, which both use methylated linker DNA as components during the DNA assembly process to introduce new BsaI sites for subsequent rounds of assembly (17,18). However, the resulting systems could only be used for iterative linear addition of DNA parts, thus limiting the speed of DNA assembly. Compared to these systems, the MetClo approach using *in vivo* methylation of methylation-switchable restriction sites outside the adaptor sequence, provides the advantages of rapid hierarchical multi-step assembly, and maintains freedom of choice for the adaptor sequences used in the assembly. In principle, similar results could be achieved by adding short methylated linker DNA as the first and last parts during the assembly process. However, this would increase the number of parts in the assembly by two, which could reduce the success rate of assembly compared to the *in vivo* vector methylation approach we have tested.

In summary, the MetClo system enables hierarchical multi-step assembly of large DNA fragments using a single type IIS restriction enzyme. This is made possible by the use of a site-specific methylase, which methylates and so blocks a methylation-switchable site for a type IIS restriction enzyme within the recipient vector. The system provides a simple restriction enzyme-based assembly method and increases the flexibility of design of assembly schemes. The BsaI-based MetClo system in particular is compatible with the existing part libraries that uses BsaI, and could be adopted as a standard assembly system.

## DATA AVAILABILITY

The plasmids have been deposited to Addgene.

## Supplementary Material

Supplementary Data
