# Supplementary material for: MetClo: methylase-assisted hierarchical DNA assembly using a single type IIS restriction enzyme
Source: Nucleic Acids Res. 2018 Jul 9;46(19):e113. doi: 10.1093/nar/gky596 (PMC6212791; doi:10.1093/nar/gky596)
Supplement: Supplementary Data [file gky596_supplemental_files.zip › 20180619 supplementary.pdf]

SUPPLEMENTARY DATA

Supplementary Tables

Table S1. Plasmids used in this study

Table S2. Assembly of 54 kb fragments using BsaI-based MoClo vectors

Supplementary Figures

Figure S1. Restriction digestion analysis for proof-of-principle DNA assembly using BsaI, BpiI or LguI-based MetClo systems

Figure S2. Assembly of a 218 kb DNA fragment using BsaI-based MetClo

Figure S3. The standard MetClo vector set

Figure S4. Scheme for assembly of DNA through an intermediate assembly stage

Figure S5. Scheme for assembly vector construction using BsaI-based MetClo

Supplementary Data File

DNA sequences of plasmids and linear DNA construct are in GenBank format in a zip file named genbank_files.zip.

Table S1. Plasmids used in this study

ID	Plasmid name	Selection marker	Note
POC1343	pMXK2_FragA	kanamycin	Insert plasmid for BsaI-based MetClo 1 kb test assembly
POC1344	pMXK2_FragB	kanamycin	Insert plasmid for BsaI-based MetClo 1 kb test assembly
POC1345	pMXK2_FragC	kanamycin	Insert plasmid for BsaI-based MetClo 1 kb test assembly
POC1346	pMXK2_FragD	kanamycin	Insert plasmid for BsaI-based MetClo 1 kb test assembly
POC1347	pMYK2_FragA	kanamycin	Insert plasmid for BpiI-based MetClo 1 kb test assembly
POC1348	pMYK2_FragB	kanamycin	Insert plasmid for BpiI-based MetClo 1 kb test assembly
POC1349	pMYK2_FragC	kanamycin	Insert plasmid for BpiI-based MetClo 1 kb test assembly
POC1350	pMYK2_FragD	kanamycin	Insert plasmid for BpiI-based MetClo 1 kb test assembly
POC1351	pMZK2_FragA	kanamycin	Insert plasmid for LguI-based MetClo 1 kb test assembly
POC1352	pMZK2_FragB	kanamycin	Insert plasmid for LguI-based MetClo 1 kb test assembly
POC1353	pMZK2_FragC	kanamycin	Insert plasmid for LguI-based MetClo 1 kb test assembly
POC1354	pMZK2_FragD	kanamycin	Insert plasmid for LguI-based MetClo 1 kb test assembly
POC1355	pMXP_A4E	ampicillin	Assembly vector for BsaI-based MetClo 1 kb test assembly
POC1356	pMYP_A5E	ampicillin	Assembly vector for BpiI-based MetClo 1 kb test assembly
POC1357	pMZP_P6T	ampicillin	Assembly vector for LguII-based MetClo 1 kb test assembly
POC1358	pMXP_test	ampicillin	Assembled plasmid from BsaI-based MetClo 1 kb test assembly
POC1359	pMYP_test	ampicillin	Assembled plasmid from BpiI-based MetClo 1 kb test assembly
POC1360	pMZP_test	ampicillin	Assembled plasmid from LguI-based MetClo 1 kb test assembly
POC1361	pMOLK_2A1	kanamycin	Insert plasmid for BsaI-based MetClo assembly of 218 kb DNA
POC1362	pMOLK_2B1	kanamycin	Insert plasmid for BsaI-based MetClo assembly of 218 kb DNA
POC1363	pMOLK_2C1	kanamycin	Insert plasmid for BsaI-based MetClo assembly of 218 kb DNA
POC1364	pMOLK_2D1	kanamycin	Insert plasmid for BsaI-based MetClo assembly of 218 kb DNA
POC1365	pMOLK_2E1	kanamycin	Insert plasmid for BsaI-based MetClo assembly of 218 kb DNA
POC1366	pMOLK_2F1	kanamycin	Insert plasmid for BsaI-based MetClo assembly of 218 kb DNA
POC1367	pMOLK_2G1	kanamycin	Insert plasmid for BsaI-based MetClo assembly of 218 kb DNA
POC1368	pMOLK_2I1	kanamycin	Insert plasmid for BsaI-based MetClo assembly of 218 kb DNA
POC1369	pMOLK_2A2	kanamycin	Insert plasmid for BsaI-based MetClo assembly of 218 kb DNA
POC1370	pMOLK_2B2	kanamycin	Insert plasmid for BsaI-based MetClo assembly of 218 kb DNA
POC1371	pMOLK_2C2	kanamycin	Insert plasmid for BsaI-based MetClo assembly of 218 kb DNA
POC1372	pMOLK_2D2	kanamycin	Insert plasmid for BsaI-based MetClo assembly of 218 kb DNA
POC1373	pMOBK_2E2	kanamycin	Insert plasmid for BsaI-based MetClo assembly of 218 kb DNA
POC1374	pMOLK_2F2	kanamycin	Insert plasmid for BsaI-based MetClo assembly of 218 kb DNA
POC1375	pMOLK_2G2	kanamycin	Insert plasmid for BsaI-based MetClo assembly of 218 kb DNA
POC1376	pMOLK_2I2	kanamycin	Insert plasmid for BsaI-based MetClo assembly of 218 kb DNA
POC1377	pMOLK_2A3	kanamycin	Insert plasmid for BsaI-based MetClo assembly of 218 kb DNA
POC1378	pMOLK_2B3	kanamycin	Insert plasmid for BsaI-based MetClo assembly of 218 kb DNA
POC1379	pMOLK_2C3	kanamycin	Insert plasmid for BsaI-based MetClo assembly of 218 kb DNA
POC1380	pMOBK_2D3	kanamycin	Insert plasmid for BsaI-based MetClo assembly of 218 kb DNA
POC1381	pMOLK_2E3	kanamycin	Insert plasmid for BsaI-based MetClo assembly of 218 kb DNA
POC1382	pMOLK_2F3	kanamycin	Insert plasmid for BsaI-based MetClo assembly of 218 kb DNA
POC1383	pMOLK_2G3	kanamycin	Insert plasmid for BsaI-based MetClo assembly of 218 kb DNA
POC1384	pMOLK_2I3	kanamycin	Insert plasmid for BsaI-based MetClo assembly of 218 kb DNA
POC1385	pMOLK_2A4	kanamycin	Insert plasmid for BsaI-based MetClo assembly of 218 kb DNA
POC1386	pMOLK_2B4	kanamycin	Insert plasmid for BsaI-based MetClo assembly of 218 kb DNA
POC1387	pMOLK_2C4	kanamycin	Insert plasmid for BsaI-based MetClo assembly of 218 kb DNA
POC1388	pMOLK_2D4	kanamycin	Insert plasmid for BsaI-based MetClo assembly of 218 kb DNA
POC1389	pMXBG_3A1	gentamicin	Assembled plasmid from stage 1 BsaI-based MetClo assembly of 218 kb DNA
POC1390	pMXBG_3I1	gentamicin	Assembled plasmid from stage 1 BsaI-based MetClo assembly of 218 kb DNA
POC1391	pMXBG_3G1	gentamicin	Assembled plasmid from stage 1 BsaI-based MetClo assembly of 218 kb DNA
POC1392	pMXBG_3F1	gentamicin	Assembled plasmid from stage 1 BsaI-based MetClo assembly of 218 kb DNA
POC1393	pMXBK_4A1	kanamycin	Final assembled plasmid from stage 2 BsaI-based MetClo assembly of 218 kb DNA
POC1394	pMXBG_A7I	gentamicin	Assembly vector for stage 1 BsaI-based MetClo assembly of 218 kb DNA
POC1395	pMXBG_I7G	gentamicin	Assembly vector for stage 1 BsaI-based MetClo assembly of 218 kb DNA
POC1396	pMXBG_G7F	gentamicin	Assembly vector for stage 1 BsaI-based MetClo assembly of 218 kb DNA
POC1397	pMXBG_F7E	gentamicin	Assembly vector for stage 1 BsaI-based MetClo assembly of 218 kb DNA
POC1398	pMXBK_A7E	kanamycin	Assembly vector for stage 2 BsaI-based MetClo assembly of 218 kb DNA
POC1399	pMOP_BsaINC	ampicillin	Test plasmid for verification of the blocking of overlapping BsaI/M.Osp807II site by M.Osp807II
POC1400	pMOP_BpiINC	ampicillin	Test plasmid for verification of the blocking of overlapping BpiI/M2.NmeMC58II site by M2.NmeMC58II
POC1401	pMOP_LguINC	ampicillin	Test plasmid for verification of the blocking of overlapping LguI/M.XmnI site by M.XmnI
POC1402	pMOBK-zeo-arsb-MOsp	kanamycin	Low copy plasmid for M.Osp807II expression
POC1403	pMOBK-zeo-arsb-M2Nme	kanamycin	Low copy plasmid for M2.NmeMC58II expression
POC1404	pMOBK-zeo-arsb-MXmnI	kanamycin	Low copy plasmid for M.XmnI expression
POC1411	pMOBG_A3I	gentamicin	Assembly vector for BsaI-based MoClo assembly of 54 kb DNA
POC1412	pMOBG_I3G	gentamicin	Assembly vector for BsaI-based MoClo assembly of 54 kb DNA
POC1413	pMOBG_G3F	gentamicin	Assembly vector for BsaI-based MoClo assembly of 54 kb DNA
POC1414	pMOBG_F3E	gentamicin	Assembly vector for BsaI-based MoClo assembly of 54 kb DNA
POC1415	pMOBG_3A1	gentamicin	Assembled plasmid from BsaI-based MoClo assembly of 54 kb DNA
POC1416	pMOBG_3I1	gentamicin	Assembled plasmid from BsaI-based MoClo assembly of 54 kb DNA
POC1417	pMOBG_3G1	gentamicin	Assembled plasmid from BsaI-based MoClo assembly of 54 kb DNA
POC1418	pMOBG_3F1	gentamicin	Assembled plasmid from BsaI-based MoClo assembly of 54 kb DNA
POC12355	pMXLC_pFa	chloramphenicol	Low copy assembly vector from the standard MetClo assembly vector set
POC12356	pMXLC_aFb	chloramphenicol	Low copy assembly vector from the standard MetClo assembly vector set
POC12357	pMXLC_bFc	chloramphenicol	Low copy assembly vector from the standard MetClo assembly vector set
POC12358	pMXLC_cFd	chloramphenicol	Low copy assembly vector from the standard MetClo assembly vector set
POC12359	pMXLC_dFe	chloramphenicol	Low copy assembly vector from the standard MetClo assembly vector set
POC12362	pMXLC_aFq	chloramphenicol	Low copy assembly vector from the standard MetClo assembly vector set
POC12363	pMXLC_bFq	chloramphenicol	Low copy assembly vector from the standard MetClo assembly vector set
POC12364	pMXLC_cFq	chloramphenicol	Low copy assembly vector from the standard MetClo assembly vector set
POC12365	pMXLC_dFq	chloramphenicol	Low copy assembly vector from the standard MetClo assembly vector set
POC12366	pMXLC_eFq	chloramphenicol	Low copy assembly vector from the standard MetClo assembly vector set
POC12367	pMXLK_pFa	kanamycin	Low copy assembly vector from the standard MetClo assembly vector set
POC12368	pMXLK_aFb	kanamycin	Low copy assembly vector from the standard MetClo assembly vector set
POC12369	pMXLK_bFc	kanamycin	Low copy assembly vector from the standard MetClo assembly vector set
POC12370	pMXLK_cFd	kanamycin	Low copy assembly vector from the standard MetClo assembly vector set
POC12371	pMXLK_dFe	kanamycin	Low copy assembly vector from the standard MetClo assembly vector set
POC12374	pMXLK_aFq	kanamycin	Low copy assembly vector from the standard MetClo assembly vector set
POC12375	pMXLK_bFq	kanamycin	Low copy assembly vector from the standard MetClo assembly vector set
POC12376	pMXLK_cFq	kanamycin	Low copy assembly vector from the standard MetClo assembly vector set
POC12377	pMXLK_dFq	kanamycin	Low copy assembly vector from the standard MetClo assembly vector set
POC12378	pMXLK_eFq	kanamycin	Low copy assembly vector from the standard MetClo assembly vector set
POC12379	pMXLP_pFa	ampicillin	Low copy assembly vector from the standard MetClo assembly vector set
POC12380	pMXLP_aFb	ampicillin	Low copy assembly vector from the standard MetClo assembly vector set
POC12381	pMXLP_bFc	ampicillin	Low copy assembly vector from the standard MetClo assembly vector set
POC12382	pMXLP_cFd	ampicillin	Low copy assembly vector from the standard MetClo assembly vector set
POC12383	pMXLP_dFe	ampicillin	Low copy assembly vector from the standard MetClo assembly vector set
POC12386	pMXLP_aFq	ampicillin	Low copy assembly vector from the standard MetClo assembly vector set
POC12387	pMXLP_bFq	ampicillin	Low copy assembly vector from the standard MetClo assembly vector set
POC12388	pMXLP_cFq	ampicillin	Low copy assembly vector from the standard MetClo assembly vector set
POC12389	pMXLP_dFq	ampicillin	Low copy assembly vector from the standard MetClo assembly vector set
POC12390	pMXLP_eFq	ampicillin	Low copy assembly vector from the standard MetClo assembly vector set
POC12391	pMXBC_pFa	chloramphenicol	Single copy assembly vector from the standard MetClo assembly vector set
POC12392	pMXBC_aFb	chloramphenicol	Single copy assembly vector from the standard MetClo assembly vector set
POC12393	pMXBC_bFc	chloramphenicol	Single copy assembly vector from the standard MetClo assembly vector set
POC12394	pMXBC_aFq	chloramphenicol	Single copy assembly vector from the standard MetClo assembly vector set
POC12395	pMXBC_bFq	chloramphenicol	Single copy assembly vector from the standard MetClo assembly vector set
POC12396	pMXBC_cFq	chloramphenicol	Single copy assembly vector from the standard MetClo assembly vector set
POC12397	pMXBK_pFa	kanamycin	Single copy assembly vector from the standard MetClo assembly vector set
POC12398	pMXBK_aFb	kanamycin	Single copy assembly vector from the standard MetClo assembly vector set
POC12399	pMXBK_bFc	kanamycin	Single copy assembly vector from the standard MetClo assembly vector set
POC12400	pMXBK_aFq	kanamycin	Single copy assembly vector from the standard MetClo assembly vector set
POC12401	pMXBK_bFq	kanamycin	Single copy assembly vector from the standard MetClo assembly vector set
POC12402	pMXBK_cFq	kanamycin	Single copy assembly vector from the standard MetClo assembly vector set
POC12403	pMXBP_pFa	ampicillin	Single copy assembly vector from the standard MetClo assembly vector set
POC12404	pMXBP_aFb	ampicillin	Single copy assembly vector from the standard MetClo assembly vector set
POC12405	pMXBP_bFc	ampicillin	Single copy assembly vector from the standard MetClo assembly vector set
POC12406	pMXBP_aFq	ampicillin	Single copy assembly vector from the standard MetClo assembly vector set
POC12407	pMXBP_bFq	ampicillin	Single copy assembly vector from the standard MetClo assembly vector set
POC12408	pMXBP_cFq	ampicillin	Single copy assembly vector from the standard MetClo assembly vector set

Table S2. Assembly of 54 kb fragments using BsaI-based MoClo vectors

Plasmid	Vector	Inserts							Size	Success rate*
pMOBG_3A1	pMOBG_A3I	pMOLK_2A1	pMOLK_2B1	pMOLK_2C1	pMOLK_2D1	pMOLK_2E1	pMOLK_2F1	pMOLK_2G1	54 kb	83% (5/6)
pMOBG_3I1	pMOBG_I3G	pMOLK_2I1	pMOLK_2A2	pMOLK_2B2	pMOLK_2C2	pMOLK_2D2	pMOBK_2E2	pMOLK_2F2	55 kb	33% (2/6)
pMOBG_3G1	pMOBG_G3F	pMOLK_2G2	pMOLK_2I2	pMOLK_2A3	pMOLK_2B3	pMOLK_2C3	pMOBK_2D3	pMOLK_2E3	54 kb	67% (4/6)
pMOBG_3F1	pMOBG_F3E	pMOLK_2F3	pMOLK_2G3	pMOLK_2I3	pMOLK_2A4	pMOLK_2B4	pMOLK_2C4	pMOLK_2D4	54 kb	83% (5/6)

* calculated based on the white colonies selected following blue-white screening

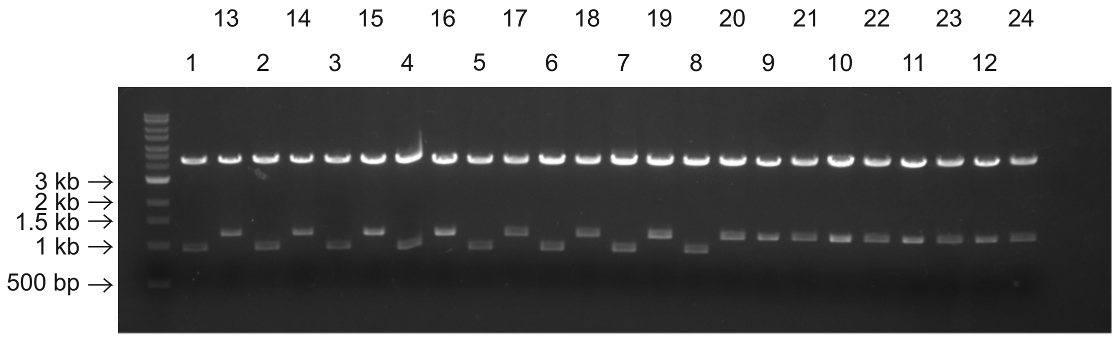

Figure S1. Restriction digestion analysis for proof-of-principle DNA assembly using BsaI, BpiI or LguI-based MetClo systems

8 clones each of assembled plasmids for proof-of-principle MetClo assembly using BsaI (Lane 1-8), BpiI (Lane 9-16) or LguI (Lane 17-24) were verified by restriction digestion using the corresponding restriction enzymes. The assembled DNA of the correct size was released from all the clones using the corresponding restriction enzyme.

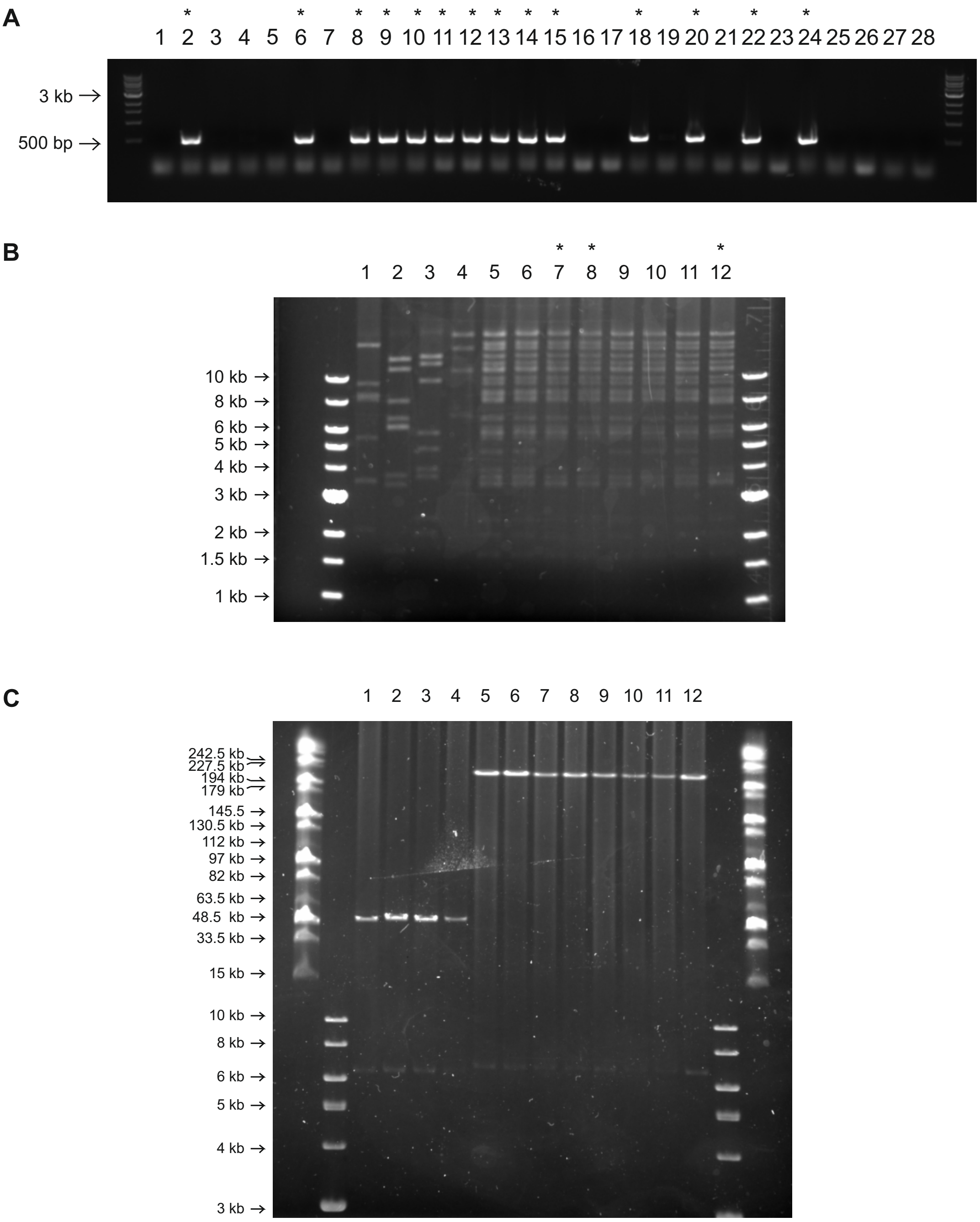

Figure S2. Assembly of a 218 kb DNA fragment using BsaI-based MetClo

A. PCR screening of 27 white clones from stage two MetClo assembly of the 218 kb DNA fragment (Lane 1-27) and negative control (Lane 28). The positive clones from the PCR screening are indicated with an asterisk (*). **B**. Restriction digestion analysis of 8 positive clones from the PCR screening (clones #2, 6, 8, 9, 10, 11, 12, 13 from **A**) (Lane 5-12), along with the four 54 kb clones used as insert plasmids for stage two assembly (Lane 1-4) by XhoI. The clones with a restriction pattern consistent with the predicted pattern are indicated with an asterisk (*). **C**. Pulsed field gel electrophoresis analysis of 8 positive clones from the PCR screening (clones #2, 6, 8, 9, 10, 11, 12, 13 from **A**) (Lane 5-12), along with the four 54 kb clones used as insert plasmids for stage two assembly (Lane 1-4) digested with BsaI. All the 8 PCR positive clones showed the correct 218 kb band.

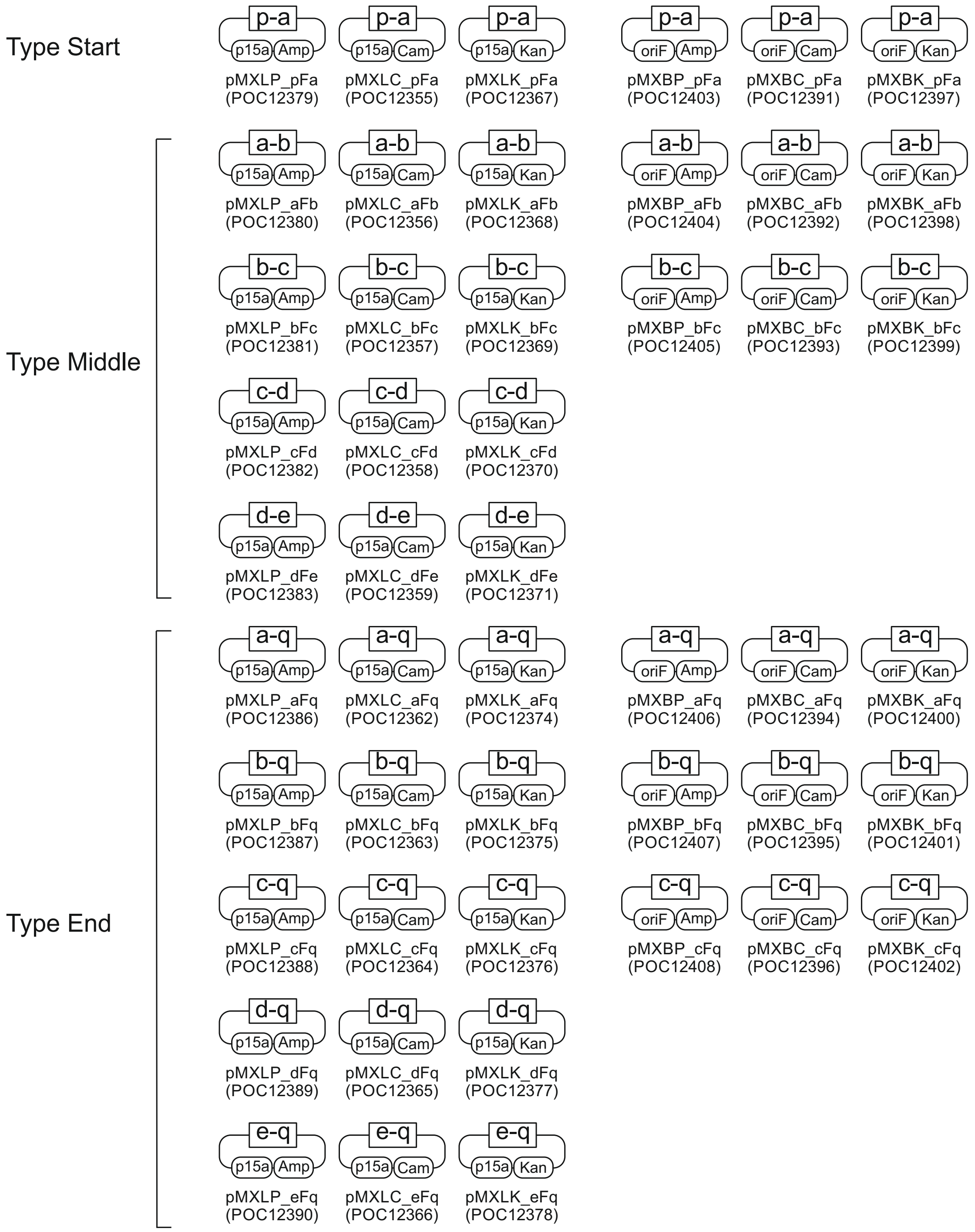

Figure S3. The standard MetClo vector set

The vector set contains 30 low copy vectors with a p15a replication origin and either ampicillin (Amp), kanamycin (Kan) or chloramphenicol (Cam) selection marker, and a LacZα negative selection marker flanked by different adaptor sequences sufficient for the assembly of up to 6 fragments in a single reaction. The vector set also contains 18 low copy vectors with an F replication origin (oriF), one of the three antibiotic selection markers, and a fragment containing a ColE1 replication origin and a LacZα negative selection marker flanked by different adaptor sequences sufficient for assembly of up to 4 fragments in a single reaction.

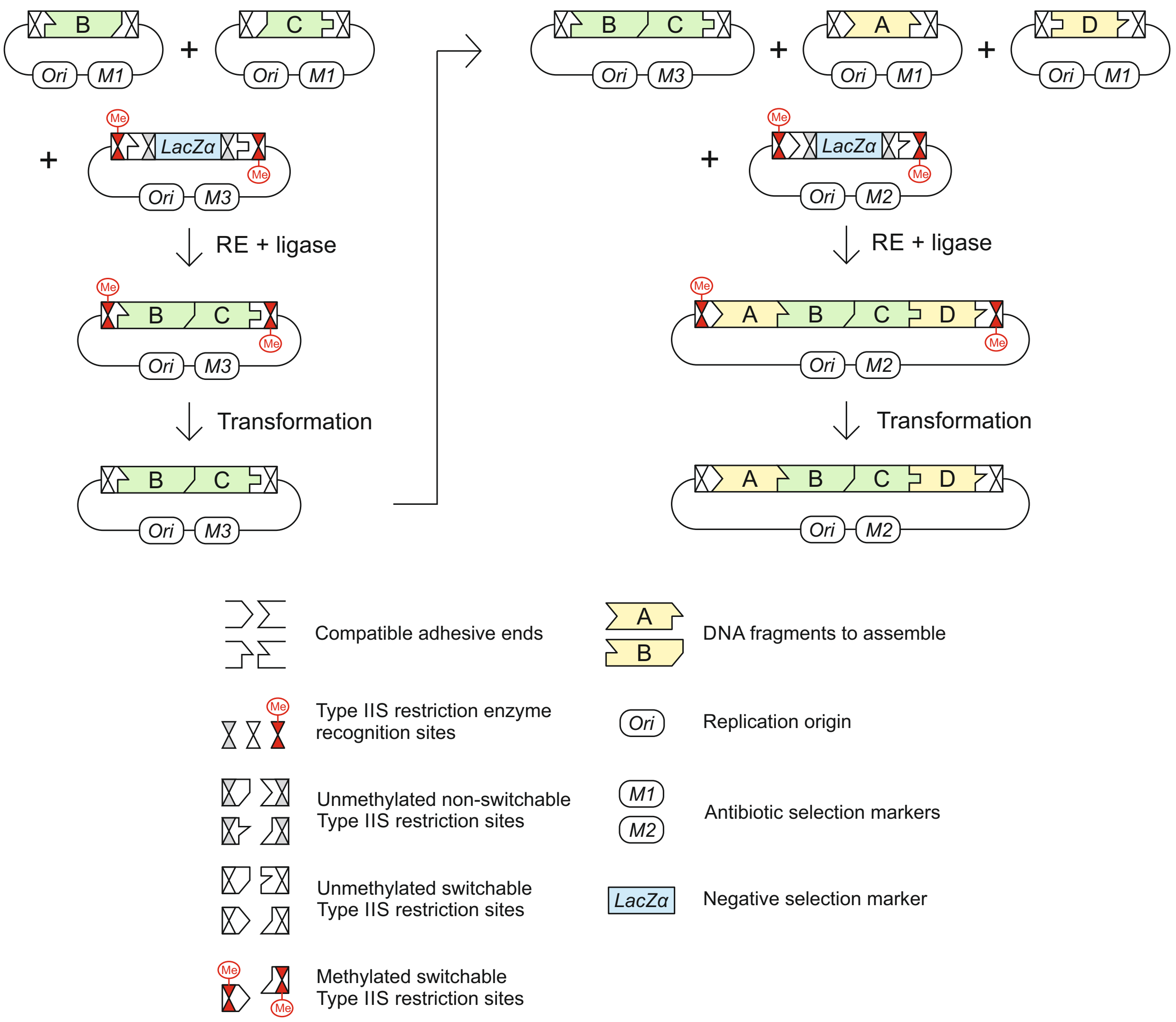

Figure S4. Scheme for assembly of DNA through an intermediate assembly stage

The diagram shows the scheme for assembly of four DNA fragments (Fragments A, B, C and D) into a single fragment (Fragment ABCD) through an intermediate stage of DNA assembly. In contrast to the standard MetClo assembly scheme (Figure 3) that assembles the four fragments in a single reaction, this scheme involves an intermediate assembly stage by sub-assembly of fragments B and C into an intermediate assembly vector that carries a selection marker (M3) different from the one for level 1 DNA inserts (M1 carried by fragments A, B, C and D) and for level 2 assembled DNA fragment (M2 carried by fragments ABCD). The sub-assembly scheme offers more choices in the routes of DNA assembly, and increases the reusability of intermediate assembled DNA fragments.

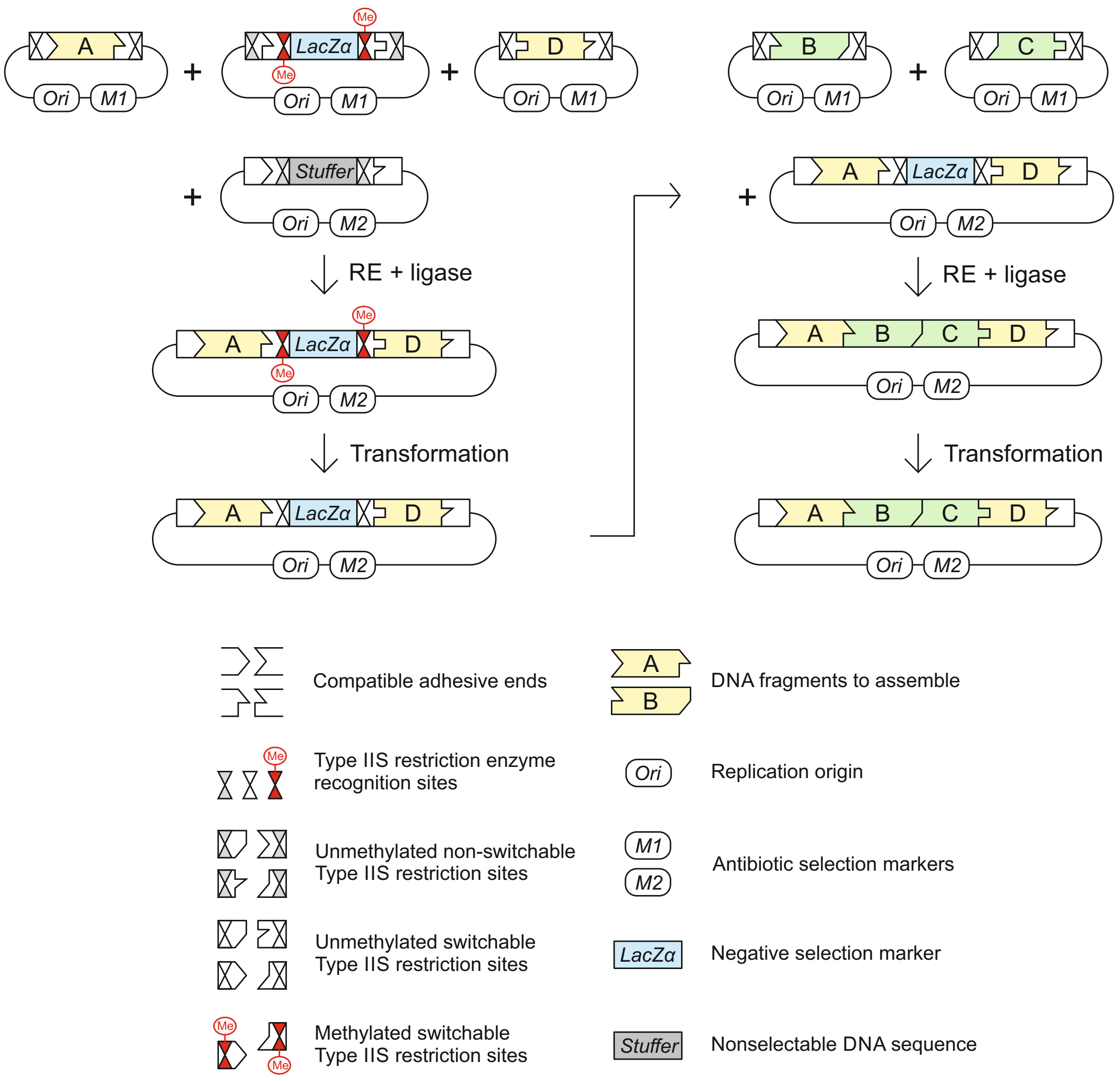

Figure S5. Scheme for assembly vector construction using BsaI-based MetClo

The diagram shows the scheme for linear addition of DNA parts for construction of assembly vectors carrying existing functional genetic elements (Fragments A and D). Starting with an assembly vector that carries a nonselectable stuffer DNA fragment flanked by nonswitchable type IIS restriction sites, two DNA fragments (Fragments A and D) are first assembled with a LacZα selection marker into this vector. The LacZα selection marker is flanked by a specially designed head-to-head arrangement of type IIS restriction sites, such that the inner sites are methylation-switchable and the outer sites are non-switchable. Preparation of the LacZα insert plasmid in an *E. coli* strain expressing the switch methylase switches off the inner pairs of type IIS restriction sites specifically. One-pot assembly of the LacZα insert with other inserts prepared from the normal *E. coli* strain (Fragments A and D) results in an assembled DNA fragment refractory to restriction by the type IIS restriction enzyme used for DNA assembly. Transformation of the assembled DNA into a normal *E. coli* strain that lacks the switch methylase activity results in demethylation of the methylation-switchable type IIS restriction sites flanking the LacZα fragment. As a result, the assembled DNA can then be used as an assembly vector for addition of new DNA fragments (Fragments B and C) in place of the LacZα selection marker in the next round of DNA assembly. The net outcome is linear addition of DNA fragments into the assembly vector (Fragments A and D, then fragments B and C). The scheme is useful for construction of final stage assembly vectors that carry common functional genetic elements, such as mammalian or plant selection markers.
